# Differences between patients' and clinicians' report of sleep disturbance: a field study in mental health care in Norway

**DOI:** 10.1186/1471-244X-11-186

**Published:** 2011-11-23

**Authors:** Håvard Kallestad, Bjarne Hansen, Knut Langsrud, Torleif Ruud, Gunnar Morken, Tore C Stiles, Rolf W Gråwe

**Affiliations:** 1 St. Olav's University Hospital, Division of Psychiatry, Department of Research and Development. Trondheim, Norway; 2 Norwegian University of Science and Technology, Faculty of Medicine, Department of Neuroscience. Trondheim, Norway; 3 St. Olav's University Hospital, Division of Psychiatry, Department of Østmarka. Trondheim, Norway; 4 Akershus University Hospital, Division of Mental Health Services. Norway; 5 University of Oslo, Faculty of Medicine, Institute of Clinical Medicine. Oslo, Norway; 6 Norwegian University of Science and Technology, Faculty of Social Sciences and Technology Management, Department of Psychology. Trondheim, Norway; 7 University of Oslo, Institute of Clinical Medicine, Norwegian Centre for Addiction Research, Oslo, Norway; 8 Drug and Alcohol Treatment Health Trust Central Norway. Department of Research and Development, Trondheim, Norway

## Abstract

**Background:**

The aims of the study was to assess the prevalence of diagnosed insomnia and the agreement between patient- and clinician-reported sleep disturbance and use of prescribed hypnotic medication in patients in treatment for mental disorders.

**Methods:**

We used three cross-sectional, multicenter data-sets from 2002, 2005, and 2008. Data-set 1 included diagnostic codes from 93% of all patients receiving treatment in mental health care in Norway (*N *= 40261). Data-sets 2 (*N *= 1065) and 3 (*N *= 1181) included diagnostic codes, patient- and clinician-reported sleep disturbance, and use of prescribed hypnotic medication from patients in 8 mental health care centers covering 10% of the Norwegian population.

**Results:**

34 patients in data-set 1 and none in data-sets 2 and 3 had a diagnosis of insomnia as a primary or comorbid diagnosis. In data-sets 2 and 3, 42% and 40% of the patients reported sleep disturbance, whereas 24% and 13% had clinician-reported sleep disturbance, and 7% and 9% used hypnotics. Patients and clinicians agreed in 29% and 15% of the cases where the patient or the clinician or both had reported sleep disturbance. Positive predictive value (PPV) of clinicians' evaluations of patient sleep disturbance was 62% and 53%. When the patient reported sleep disturbance as one of their most prominent problems PPV was 36% and 37%. Of the patients who received hypnotic medication, 23% and 29% had neither patient nor clinician-rated sleep disturbance.

**Conclusion:**

When patients meet the criteria for a mental disorder, insomnia is almost never diagnosed, and sleep disturbance is imprecisely recognized relative to the patients' experience of sleep disturbance.

## Background

Sleep disturbance is likely to be a core feature across several mental disorders [1,2]. In patients with depression, sleep disturbance predicts poorer treatment outcome and is associated with more suicide attempts [3,4]. Moreover, 40%-70% of patients successfully treated for depression still experience sleep disturbance after treatment [3,5], and these patients may be at a higher risk of relapses into new episodes [6]. This challenges the assumption that the sleep disturbance will also be improved once the primary mental disorder is treated. It may be more adequate to assume that there is a need for specific treatment for sleep disturbance for these patients.

Adequate management of insomnia starts with recognition and diagnostic assessment. There is a risk, however, that insomnia could become trivialized in mental health care because it is both a diagnosis as well as a symptom of several mental disorders. But although many may share the impression that insomnia is under-recognized by clinicians [7,8], the actual data available from medical practice is limited and ambiguous. Two studies have confirmed that sleep is seldom documented in medical charts in general hospitals [9] or on general medical services [10], whereas a recent large-scale study concluded that sleep disturbance is adequately recognized by general practitioners [11]. Only one study has been conducted to test if sleep disturbance is clinically recognized in patients with mental disorders [12]. The authors found that 80 of 100 patients on a psychiatric consultation service had sleep disorders but disturbed sleep was not mentioned in the records of 54% of these patients. However, the study was conducted in the early 1980s and had limited external validity. The state of sleep in psychiatry might be different 30 years later. Thus, even though 50% to 80% of patients with mental disorders have sleep disturbance [13], there is currently limited empirical knowledge about diagnostic assessment, clinical recognition and treatment for this large group of patients.

As the DSM and the ICD will be revised during the next years there is currently a need for data that can inform this revision process [14]. The primary users of these diagnostic systems are mental health care personnel and general medical clinicians and the classifications should therefore be meaningful for these groups [14]. A basic test of clinical utility is to explore whether clinicians in mental health care use the insomnia diagnosis. However, because insomnia is also a symptom of several mental disorders, there is a possibility that clinicians could recognize and treat sleep disturbance without diagnosing it.

The aims of this study were therefore to examine whether insomnia is diagnosed and whether sleep disturbance is recognized and treated in patients with mental disorders. We used three large, cross sectional, multicenter data-sets for this purpose.

## Methods

Data-set 1 included demographic information and clinical diagnostic codes from an estimated 93% of all patients receiving treatment in mental health care, both private practice and public health care, in Norway during two weeks in 2008.

In addition to demographic and clinical diagnostic assessments, data-sets 2 and 3 also included both clinicians' and patients' report of sleep disturbance and prescriptions of hypnotic medication. Data-sets 2 and 3 were conducted at eight public mental health centers during eight weeks in 2002 and four weeks in 2005, respectively. The centers were selected to be representative for the country as a whole and the catchment areas covered about 10% of the Norwegian population. The centers covered both rural and urban areas as well as northern and southern parts of the country. The data collections were commissioned by the Norwegian Department of Health to evaluate the state of the National Mental Health Care System and were not designed specifically to evaluate sleep disturbance in patients with mental disorders.

Data-sets 1, 2 and 3 were used to assess the prevalence of clinically diagnosed insomnia. Data-sets 2 and 3 were also used to assess the agreement between clinician and patient reported sleep disturbance and prescription of hypnotic medication. The analyses were replicated in the data-sets to test the reliability of the findings.

### Participants

Data-set 1 included 40 261 patients. Patients were between 18 and 103 years old. See table 1 for demographic data.

**Table 1:** Sample characteristics in the three data-sets included in the study.

	Data-set 1 (*N = *40261)		Data-set 2 (*N = *1065)		Data-set 3 (*N = *1181)	
Age, mean (s.d.)	40.2	(14.2)	39.4	(12.1)	39.6	(12.0)

	*N*	*(%)*	*n *	*(%)*	*n *	*(%)*
Gender						
Female	24702	(61.4)	674	(63.3)	745	(63.1)
Male	14598	(36.3)	382	(35.9)	407	(34.5)
Type of treatment						
Outpatient	31627	(78.6)	903	(84.8)	911	(77.1)
Day treatment	1801	(4.5)	66	(6.2)	129	(10.9)
Inpatient	4079	(10.1)	76	(7.1)	138	(11.7)

In data-set 2, a total of 3 497 patients were enrolled and 1 065 patients (30%) were included in the current study as they agreed to have their self-report linked to their clinician's diagnoses and symptom reports and treatment. Patients were between 18 and 85 years old.

In data-set 3, a total of 3041 patients were enrolled and 1181 patients (39%) were included as they agreed to have their self-report linked to their clinician's diagnoses and symptom reports and treatment. Patients were between 18 and 82 years old.

### Assessments

#### Data collection

All data collections were commissioned by the Norwegian Department of Health and conducted by an independent research institution, SINTEF Technology and Society.

#### Diagnosis of insomnia

Primary and one (data-set 1) or two (data-sets 2 and 3) additional ICD-10 [15] diagnoses were recorded by the clinicians in charge. The procedure for the diagnostic evaluation varied within and between clinics and mirrored clinical practice. There were missing diagnoses for 46 patients in data-set 2 and 241 in data-set 3. Patients with a diagnosis of F51.0 Non-organic insomnia as either a primary, secondary or tertiary diagnosis were categorized as having a diagnosed insomnia. See table 2 for diagnostic criteria.

**Table 2:** ICD-10 diagnostic criteria for F51.0 Non-organic Insomnia.

Symptom	Individual complaints of difficulty falling asleep, difficulty maintaining sleep, or non-refreshing sleep.
Duration	At least three days a week for at least one month.
Consequence	The sleep disturbance results in marked personal distress or interference with personal functioning in daily living.
Comorbidity	If other mental disturbances are present, Insomnia should be coded as an additional disturbance if either a) sleep disturbance is one of the patient's most prominent complaints, or b) if the sleep disturbance also occurs in periods without symptoms of other mental disturbance.
Exclusion	There is no known causative organic factor. If the sleep disturbance only occurs during episodes of other mental disturbance, the primary mental disturbance should be coded.

#### Patient-reported sleep disturbance

Patients completed the Symptom Checklist - 25 (SCL-25) in data-set 2 and the Symptom Checklist - 10 in data-set 3 (SCL-10). Both are short versions of the SCL-90-R and the Norwegian translations have satisfactory validity and reliability [16]. The SCL-25 consists of 25 items, and the SCL-10 consists of 10 items, describing psychiatric symptoms rated on a 4-point scale (1 = not at all, 4 = very severe). All items on the SCL-10 are present in the SCL-25.

Three items measure sleep disturbance on the SCL-90R. On the SCL-25 and the SCL-10, these three have been reduced to one item measuring the severity of sleep disturbance. This item was used to assess patient rated level of sleep disturbance. Patients who scored 3 or 4 (quite severe or very severe) on this item were categorized as having patient-rated sleep disturbance.

#### Clinician-reported sleep disturbance

The clinicians used the Health of Nations Outcome Scales (HoNOS) to evaluate the patients. The HoNOS is a 12-item instrument measuring behavior, impairment, symptoms and social functioning for patients with mental disorders [17]. HoNOS item 8 requires the clinicians to identify mental or behavioral problems from a list of 10: phobic, anxiety, obsessive-compulsive, stress, dissociative, somatoform, eating, sleep disturbance, sexual, or other. The clinicians reported the three most prominent problems in data-set 2 and the most prominent problem in data-set 3 on HoNOS item 8. The HoNOS item 8 was rated on a binary scale for the current study (present or not present). Patients who the clinicians indicated had a sleep disturbance on HoNOS Item 8 were categorized as having clinician-rated sleep disturbance.

#### Hypnotic medication

Patients who were prescribed one or more of the following medications: Zopiclone, Zolpidem, Nitrazepam, Flunitrazepam, Trimeprazime and Promethazine.

### Statistical analyses

The clinicians' accuracy in recognizing the patients' sleep disturbance was reported as positive and negative predictive value. The following definitions were used.

True Positive (TP) means both the patient and clinician had reported sleep disturbance; False Positive (FP) means the patient had not reported sleep disturbance but the clinician had; False Negative (FN) means that the patient had reported sleep disturbance but the clinician had not; True Negative (TN) means the both the patient and the clinician had not reported sleep disturbance.

Positive predictive value was calculated using the equation: TP/(TP + FP). This indicates the proportion of the clinicians' evaluations of sleep disturbance that were correct, i. e. similar to patient experience. Negative predictive value was calculated using the equation: TN/(FN + TN). This indicates the proportion of clinicians' evaluations of no sleep problem that were correct.

Differences between data-set 2 and 3 were calculated using Mann-Whitney U-tests. Diagnostic differences in agreement were calculated using Chi-squared analyses. Because of the large sample size and multiple tests, level of statistical significance was set to *p *< 0.001.

### Ethics

The three studies were approved by The Regional Committee for Medical Research Ethics, Middle Norway.

## Results

### Diagnostic prevalence

In data-set 1, 34 of 40 621 patients (0.08%, [95% CI: 0.05% - 0.11%]) had received an ICD-10 diagnosis of insomnia. In data-sets 2 and 3 no patients had received an ICD-10 diagnosis of insomnia.

### Prevalence of patient and clinician-reported sleep disturbance and use of hypnotics

Table 3 indicates the prevalence of patient and clinician-reported sleep disturbance and prescription of hypnotics in data-sets 2 and 3.

**Table 3:** Prevalence of sleep disturbance in patients with mental disorders.

	*Data-set 2* (*N = *1065)			*Data-set 3* (*N = *1181)		
**Sleep disturbance**	***n***	***%***	***95% CI ***	***n***	***%***	***95% CI***

Patient-reported	447	42	39 - 45	475	40	37 - 43
Clinician-reported*	255	24	21 - 27	152	13	11 - 15
Hypnotic medication	73	7	5 - 8	106	9	7 - 11

* Please note that a more conservative measure of clinician-reported sleep disturbance was used in data-set 3 compared to data-set 2.

233 patients (22% [19% - 24%]) in data-set 2 and 299 patients (25% [23% - 28%]) in data-set 3, reported sleep disturbance to be one of the most prominent symptoms, as indicated by a score of 3 or above on the SCL sleep item and scores of less than, or equal to, the score of sleep disturbance on the remaining 24 symptoms on the SCL-25 (data-set 2) or 9 symptoms on the SCL-10 (data-set 3). In addition, 331 patients (31%, [28% - 34%]) in data-set 2 and 416 patients (35% [32% - 38%]) in data-set 3, reported sleep difficulties of mild symptom severity (score of 2 on the SCL sleep item).

There were more patients with clinician-rated sleep disturbance in data-set 2 compared to data-set 3 (*p *< 0.001). There were no differences between the two data-sets in patient-rated sleep disturbance and prescription of hypnotic medication.

### Demographic differences

There were no differences between patients in any of the sleep disturbance categories or prescription of hypnotic medication and other patients in age, gender, type of treatment, or between the eight different clinics in either data-set 2 or 3.

### Agreement

Of the patients with either patient- or clinician rated sleep disturbance, patients and clinicians agreed in 29% (25% - 33%) of the cases in data-set 2 and 15% (12% - 18%) in data-set 3.

The average positive predictive value (PPV) of clinicians' evaluations of the patients' sleep disturbance was 62% (58% - 66%) in data-set 2 and 53% (49% - 57%) in data-set 3. Average negative predictive value (NPV) was 64% (60% - 68%) in data-set 2 and 61% (57% - 65%) in data-set 3.

To test if clinicians had higher precision rate if the patient experienced sleep disturbance as one of their most prominent symptoms, we also calculated PPV and NPV for patients with self-reported sleep disturbance as one of the most prominent symptoms. For these patients, PPV was 36% (30% - 42%) in data-set 2 and 37% (32% - 42%) in data-set 3. NPV was 83% (78% - 87%) in data-set 2 and 76% (71% - 81%) in data-set 3.

Among the patients who were prescribed hypnotic medication, 55% (44% - 66%) had a clinician reported sleep disturbance in data-set 2 and 26% (18% - 34%) in data-set 3. In addition, 23% (13% - 33%) in data-set 2 and 29% (20% - 38%) in data-set 3 had neither patient- nor clinician-rated sleep disturbance.

**Figure 1:**
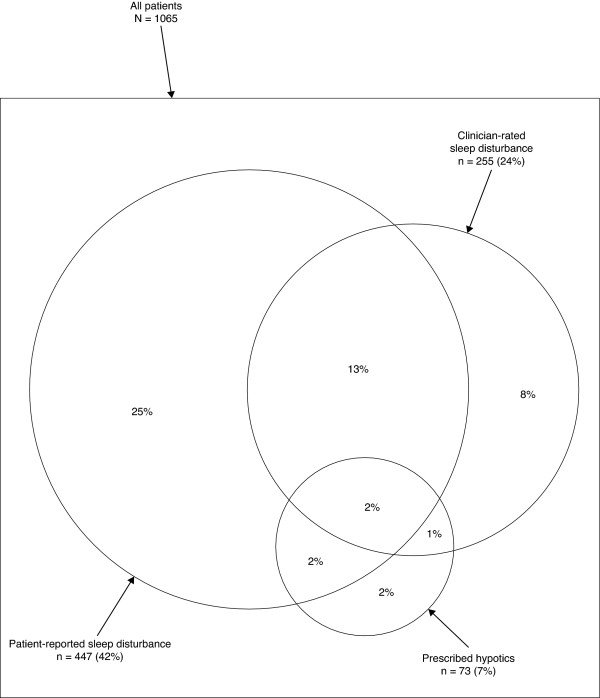
**Venn diagram describing agreement in data-set 2**. The overlap between the patients with self-reported sleep disturbance, patients with clinician reported sleep disturbance and patients who were prescribed hypnotic medication, in data-set 2.

**Figure 2:**
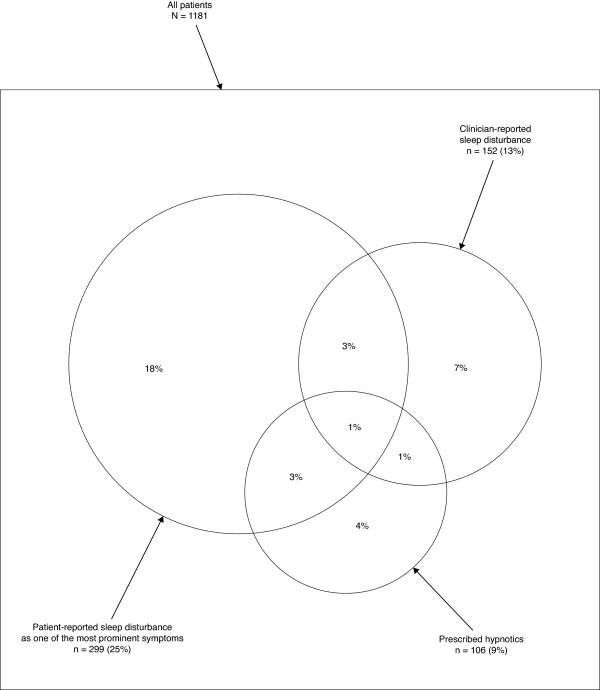
**Venn diagram describing agreement in data-set 3**. The overlap between the patients with self-reported sleep disturbance as one of the most prominent problems, patients with clinician reported sleep disturbance and patients who were prescribed hypnotic medication, in data-set 3.

The overlap between the patients with self-reported sleep disturbance, the patients with clinician-rated sleep disturbance and the patients who were prescribed hypnotic medication in data-set 2 are described in Figure 1. The overlap between patients with a self-reported sleep disturbance as one of the most prominent symptoms, patients with clinician rated sleep disturbance and patients who were prescribed hypnotic medication in data-set 3 are described in Figure 2.

Because clinicians might be more likely to assess sleep disturbance in certain disorders, we conducted *χ^2 ^*for selected diagnostic groups (substance abuse, schizophrenia, bipolar disorder, depressive episode, recurrent depressive episode, anxiety disorders, adjustment disorders and personality disorders) in chapter V of the ICD-10 to test if there were differences in agreement in these patient groups. There were no differences in agreement in any diagnostic group in data-set 2. In data-set 3, agreement was higher when the patient had a diagnosis of depressive episode (*χ^2^(1) *= 31.6, *p *≤ 0.001) compared to other patients.

## Discussion

Using three independent and nationally representative data-sets, we found that the diagnosis of insomnia was virtually non-existent in patients with mental disorders. Only 34 of 42507 patients had a diagnosis of insomnia as either a primary or a comorbid diagnosis. However, about 40% of all patients experienced severe sleep disturbance and at least 22% reported this to be one of their most prominent problems. Although they did not diagnose it, clinicians indicated that 24% of their patients had sleep disturbance as a prominent problem. We found a notable discrepancy between the clinicians' report and the patients' report of sleep disturbance. The patients and clinicians agreed in less than 30% of the cases where either the patients or clinicians had reported sleep disturbance. The probability of the patients themselves experiencing sleep disturbance when the clinician reported this, was less than 62%. This probability was surprisingly even lower when the patient experienced their sleep disturbance to be one of their most prominent symptoms, with a predictive value of less than 37%. Thus, the precision of clinician-rated sleep disturbance was close-to-chance when compared to the patient report. Of the patients who used hypnotics, 55% reported having sleep disturbance whereas 23% had neither clinician nor patient reported sleep disturbance. These findings were replicated with only minor differences in the different samples.

The diagnostic findings are at odds with findings from epidemiological studies where the prevalence has been reported to be about 100 times higher [18-20]. However, our findings are in line with two previous studies where large discrepancies between findings from epidemiological studies and diagnostic practice in clinical settings have been reported [21,22]. Although the diagnostic recommendation in the ICD-10 is to code insomnia as a comorbid disorder if it is one of the most prominent symptoms, our findings indicate that clinicians are reluctant to use these recommendations.

Underlying this may be a long-standing issue of whether insomnia should be regarded as a symptom of other disorders or as a disorder in it self. Clinicians may not find it relevant to code insomnia as a comorbid condition as they regard it as a part of the primary mental disorder. If clinicians do not find the diagnosis of insomnia relevant to their practice, this may raise concerns about the usefulness of the diagnosis in its current form. In this respect it is interesting to note that the online draft of the DSM-5 proposes to remove the diagnosis of Insomnia Related to Another Mental Disturbance and only operate with the diagnosis of Insomnia. The proposed change in the DSM reflects a change in paradigm from thinking of insomnia as a symptom to thinking of insomnia as a disorder. It recommends coding Insomnia Disorder if the criteria are fulfilled regardless of meeting criteria for other mental disorders, because determining the cause or consequence of the problem is clinically difficult, if not impossible [23].

Patient report of sleep disturbance was higher than what is found in the general population using similar measures [19], but in agreement with other studies from patients with mental disorders [13]. Hypnotic medication was, relative to previous studies [24,25], seldom prescribed to the patients in the current study. Less than 9% of all patients were prescribed such medication compared to 7% of the general population in Norway in the same period [20]. Patients with mental disorders are likely to be prescribed other medications that have effects on sleep, such as some anti-depressants or anti-psychotic medication that could make hypnotic medication less needed. Still, it is curious that at least 23% of the patients who were prescribed hypnotic medication had neither self-reported nor clinician-reported sleep disturbance. This could indicate that patients who are being prescribed hypnotics stay on hypnotics after the sleep disturbance has been treated.

The low recognition of sleep disturbance raises questions concerning education and training of health care professionals. Although a call for improved education about sleep was made in the 1980s when similar findings were reported [12], the situation does not seem to have improved. Physician training in the recognition of sleep specific symptoms has been reported to be minimal [26]. Similarly, a survey of 212 directors of graduate and internship programs of clinical psychology revealed that only 6% of the programs offered courses in sleep and most reported that their institution was ineffective in providing sleep education [27]. Moreover, doctors seldom ask about sleep in clinical interviews [28], and patients with sleep disturbance infrequently report this to their clinicians [29,30]. This makes it important for health care professionals to have knowledge about the features of sleep.

### Limitations

There is a possibility of sampling bias in data-sets 2 and 3 as about two-thirds of the original sample of patients did not agree to have their self-report linked to the clinician-report. This could be an artifact of the procedures. The patients had to specifically indicate that they wanted to have their scores linked to their clinicians' ratings, rather than having to indicate if they did not want to have their scores linked. This difference could have had a large impact on patient participation [31].

Clinicians in data-set 3 were asked to only indicate the most prominent problem the patient experienced compared to the three most prominent problems reported by clinicians in data-set 2. This gave a lower prevalence of clinician-rated sleep disturbance in data-set 3 compared to data-set 2. In both data-sets clinicians have therefore only indicated the patients who they regard as having sleep disturbance as one of their most prominent problems. The study was not primarily designed to measure sleep disturbance in mental health care and we might have found a higher prevalence of clinician-rated sleep disturbance if we had utilized a survey directly inquiring about sleep symptoms. This could have resulted in higher agreement and different predictive values for clinician's evaluations. However, our finding that even when clinicians evaluate sleep disturbance to be one of the most prominent problems, there is still a very low agreement between patients and clinicians. Especially as the predictive value of the clinicians' report was less than chance for the patients who reported sleep disturbance as one of their most prominent problems.

The use of a single item measuring sleep disturbance is another limitation. This means that it is not possible to discern if there were differences in agreement between sleep onset or sleep maintenance problems or if the patients experienced other kinds of sleep disturbance.

Finally, although we have assessed patients who received hypnotic medication, there might be a proportion of the patients who received alternate medication or non-pharmacological interventions for their sleep disturbance.

## Conclusion

When patients meet the criteria for a mental disorder, insomnia is almost never diagnosed, and sleep disturbance is imprecisely recognized relative to the patients' experience of sleep disturbance.

Our results, rather than taken as criticism of individual health care professionals, should be interpreted as a call for improved teaching and better understanding of sleep disturbance and its treatment. Moreover, the non-existence of the insomnia diagnosis in three different clinical samples may not only be a result of insufficient training, but also indicate an area where revising the ICD and DSM systems could be useful. The suggested change in the DSM-5 of coding insomnia is likely to be favorable compared to the current system and could be considered also in the ICD-11.

## Conflicts of interest

The authors declare that they have no competing interests.

## Authors' contributions

HK conceptualized the report, did the data analyses, data interpretation, and wrote the report. BH, KL, GM, and TCS conceptualized the report, did data interpretation, and critical appraisals of the report. RG and TR made the study designs, conducted the data collections, and made critical appraisals of the report. All authors read and approved the final manuscript.

## Pre-publication history

The pre-publication history for this paper can be accessed here:

http://www.biomedcentral.com/1471-244X/11/186/prepub
